# Exploring the Relationship between Ultrasonographic Measures of the Quadriceps and Knee Extensor Muscle Fitness in Endurance-Trained Individuals

**DOI:** 10.1155/2024/3415740

**Published:** 2024-04-08

**Authors:** Caleb C. Voskuil, Monique D. Dudar, Joshua C. Carr

**Affiliations:** ^1^Texas Christian University, Department of Kinesiology, Fort Worth, TX, USA; ^2^Department of Medical Education, Anne Burnett Marion School of Medicine at Texas Christian University, Fort Worth, TX, USA

## Abstract

**Background:**

B-mode ultrasonography is an accessible and cost-effective method to assess muscle size and quality through muscle thickness (MT) and echo intensity (EI), respectively. Muscle thickness and EI have demonstrated relationships with maximal strength and local muscle endurance, providing a noninvasive and efficient modality to examine muscle fitness. However, these relationships have not been quantified in the individual quadriceps muscles of habitually endurance-trained populations, which may provide information to practitioners regarding rehabilitation and performance.

**Methods:**

Twenty-three participants (males: *N* = 10; females: *N* = 13) underwent B-mode ultrasonography to assess MT, EI, and adipose tissue thickness-corrected echo intensity (cEI) in the vastus intermedius (VI), vastus lateralis (VL), and rectus femoris (RF). Muscle fitness was evaluated through maximal strength (1RM) and local muscle endurance (4 sets to failure at 50% 1RM) during dynamic knee extension. Relationships between ultrasonography outcomes and muscle fitness were examined through stepwise multiple linear regression.

**Results:**

The results indicate that VI cEI is the strongest predictor of 1RM strength (*r* = −0.643), while no ultrasonography-derived measures significantly predicted local muscle endurance.

**Conclusion:**

The study demonstrates that ultrasonography, specifically measures of cEI in the VI, has the greatest association with maximal strength in endurance-trained individuals. These findings suggest monitoring VI muscle size and quality may benefit practitioners who aim to improve knee extension strength for performance or following injury. In addition, the findings support the use of EI examinations in trained populations.

## 1. Introduction

Skeletal muscle size and muscle strength present a strong positive relationship [1–6]. The muscle size and strength relationship may be utilized to predict muscle fitness in trained populations [7], providing a noninvasive and efficient alternative to maximal testing. In addition, the relationship provides information about the ability to perform daily tasks, wherein strength is a key determinant, in injured or diseased populations in which maximal strength or endurance testing may prove difficult [8–10]. However, the quantification of muscle size can be costly when utilizing computed tomography (CT) or magnetic resonance imaging (MRI). A more affordable and accessible tool for muscle size quantification is B-mode ultrasonography [11–13]. Ultrasonography provides quantification of muscle size through the one-dimensional measure of MT or the two-dimensional measure of muscle cross-sectional area (mCSA), with reliable and comparable results to MRI and CT [14–16]. This technique also allows for individual muscle assessment, important for muscle groups such as the quadriceps wherein the vastus lateralis (VL), vastus medialis (VM), vastus intermedius (VI), and the rectus femoris (RF) synergistically produce force during knee extension. Despite this, ultrasonographic outcomes of muscle size and indices of quality of the individual quadriceps muscles and their relationships with muscle fitness are limited in resistance- or endurance-trained populations. Further investigation may provide important information to sports medicine practitioners who aim to improve muscle fitness in the quadriceps following injuries or to improve overall performance.

While muscle size has significant contributions to the production of maximal strength in the quadriceps [1, 2, 4–6, 17], the concentrations of contractile and noncontractile tissue within a muscle may affect muscle fitness. Echo intensity (EI), the greyscale color measure of an ultrasonographic image, has been hypothesized to reflect skeletal muscle quality [18]. Echo intensity values range from 0 (hypoechoic) to 255 (hyperechoic), with greater values associated with lower muscle quality [18–20]. Intramuscular fat concentrations assessed via MRI are significantly related to EI values [20–22], and increases in fat accumulation within the muscle are related to decreased force development [23, 24]. Thus, EI values have consistently demonstrated a moderate, negative relationship with knee extensor maximal strength in older adults [25–30] and demonstrate conflicting findings in younger individuals [7, 31, 32]. Recent evidence suggests that EI may be related to biceps brachii local muscle endurance in resistance-trained individuals [7], while demonstrating no relationship with local muscle endurance in the knee extensors of older adults [25] and adolescents [31]. It is reasonable to speculate the relationship between EI and local muscle endurance may be influenced by the muscle fiber composition within a muscle [7]. Type I muscle fibers present with greater intramuscular fat concentrations surrounding their muscle fiber bundles and lipid contents while providing greater fatigue resistance compared to type II fibers [33, 34]. If higher (brighter) EI values represent greater intramuscular fat [19, 20, 22], greater EI values may represent muscles with greater proportions of type I muscle fibers. Thus, habitually endurance-trained individuals, who have a greater endowment of type I muscle fibers, may demonstrate a relationship between EI and local muscle endurance. However, the relationship between EI and local muscle endurance in young, habitually endurance-trained individuals is unknown. Further examinations in habitually endurance-trained individuals may provide greater insight into the EI quantification of physiological adaptations.

Therefore, the purpose of this study is to (1) examine the relationships between skeletal muscle ultrasonography outcomes in the vastus intermedius (VI), vastus lateralis (VL), and rectus femoris (RF) with muscle fitness during knee extension in a habitually endurance-trained population and (2) determine the individual quadriceps muscle with the greatest association with maximal strength and local muscle endurance. The core features of muscle fitness are strength and endurance [35], which we investigate utilizing knee extension one repetition maximum (1RM) and repetitions performed until failure at 50% 1RM, respectively. We hypothesized that adipose tissue thickness corrected EI (cEI) [7, 20] will demonstrate the greatest associations with muscle fitness in our population. Specifically, cEI will demonstrate a negative relationship with strength and a positive relationship with local muscle endurance. We hypothesized that the RF will demonstrate the greatest association with muscle fitness compared to the other individual quadriceps muscles as it is involved in knee extension and hip flexion, movements commonly performed during endurance exercise.

## 2. Methods

### 2.1. Experimental Design

This study implements a cross-sectional design to identify associations between ultrasound-derived measurements of skeletal muscle morphology with local muscle endurance and maximal strength during dynamic knee extension in a population that habitually performs endurance exercise. Participants completed two visits to the Neuromuscular Physiology Laboratory in the Kinesiology Department at Texas Christian University, separated by 48 hours. The first visit consisted of ultrasound imaging and unilateral dynamic knee extension 1RM strength testing. The second visit consisted of participants completing a fatiguing resistance exercise protocol of four sets of knee extensions to failure with their dominant leg.

### 2.2. Participants

A total of 23 participants (female: *n* = 13; age: 21 ± 3 yrs; height: 169 ± 7 cm; mass: 61 ± 8 kg; male: *n* = 10; age: 26 ± 6 yrs; height: 177 ± 6 cm; and mass: 78 ± 11 kg) were enrolled with all participants completing the study in its entirety. To qualify for inclusion in the current study, participants must have reported performing habitual endurance exercise defined by ∼300 minutes per week of moderate to vigorous aerobic activity [35] over the last 12 months. No specific endurance activity was solely recruited, with participants reporting a wide range of modalities such as running, cycling, swimming, and triathlon as their training preference. The average reported endurance training experience for the females was 9.9 ± 4.8 years, with 7.6 ± 5.5 training hours per week, while the males reported an endurance training experience of 12.1 ± 7.7 years and 6.1 ± 4.0 training hours per week. Six of the 13 female participants indicated eumenorrhea, while the remaining 7 reported using hormonal contraceptives. All procedures were approved by the Institutional Review Board for Human Subjects at Texas Christian University (IRB# 2022-246).

## 3. Experimental Procedures

### 3.1. Instrumentation

#### 3.1.1. Ultrasonography

**Figure 1:**
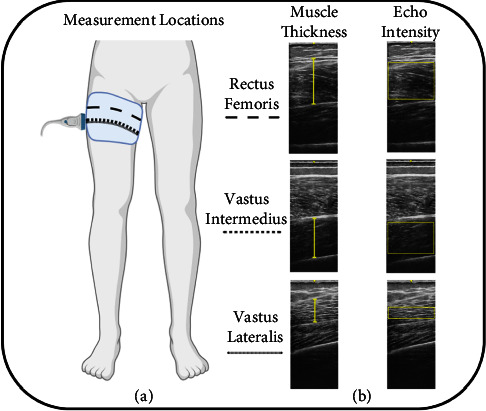
Illustrated image of the measurement locations (a) and representative images of each muscle and examples of image analysis technique (b).

Prior to strength testing, B-mode ultrasonography (GE LOGIQ E10; Software version: R9.1.2; GE Healthcare, Milwaukee, WI, USA) was used to examine muscle size and EI of the vastus lateralis (VL), vastus intermedius (VI), and rectus femoris (RF) of the participant's dominant leg. The ultrasonography images were collected utilizing a wideband linear array probe (GE 9L-RS, 3.1–10 MHz, 44 mm field of view; GE Healthcare, Milwaukee, WI, USA). The settings for the ultrasound were held consistent (frequency: 12 Hz, gain: 55 dB, and dynamic range: 72) between each participant, with changes in the depth being made only to accommodate larger muscle size and prevent image overlay in highly curved regions [36, 37]. Image acquisition was performed with the participants lying supine on an imaging table with a strap placed around their ankles to minimize extraneous movements. The images for the quadriceps muscles were taken at the location of the greatest anatomical cross-sectional area, corresponding to 40% of the distance from the anterior superior iliac spine to the lateral border of the patella for the VL and at 40% and 30% of the distance from the anterior superior iliac spine to the proximal border of the patella for the VI and RF, respectively [38].  Figure 1 shows the locations for image acquisition in addition to representative images of each muscle and examples of the image analysis.

Prior to imaging, a generous amount of water-soluble ultrasonography transmission gel was applied to the skin to enhance imaging quality. In addition, the participants were laid supine for ∼5 minutes prior to image acquisition to account for fluid shift stabilization [39]. During image acquisition, a flexible high-density foam pad was placed parallel to the sagittal acquisition plane in which the probe could be properly orientated to ensure probe tilt was held consistent between measurement locations and participants. The pressure of the probe was maintained by a skilled sonographer with ∼1.5 years of experience who ensured that the probe head was in contact with ultrasonography gel but did not deform the underlying tissue. Images were taken solely in the sagittal plane as previous work from our laboratory demonstrates that sagittal plane imaging has the strongest relationship with measures of muscle fitness in the upper limb [7]. Images were taken at each location until three scans met acceptable imaging quality, with the highest quality image analyzed through ImageJ Software (version 1.53). Each image was exported following acquisition to a portable storage device and analyzed as a JPG image.

Prior to image analysis, the researchers underwent training to minimize the influence of experience on image analysis [37]. Each image was analyzed using the consistent technique, utilizing the straight-line function to first scale image pixels to cm. The straight-line function was then utilized to determine the MT at the midpoint of the muscle on the freeze frame image. The same process was performed to generate a measure of adipose tissue thickness at the midpoint of the muscle, spanning the distance from the surface of the skin to the muscle. Using the rectangular function, a maximal region of interest was generated to include as much of the muscle as possible without including surrounding fascia to examine EI [37, 40]. The adipose tissue thickness measurements and EI were utilized to correct the value of EI for adipose tissue thickness and produce the measure of cEI using the following equation [20]:

(1) $$\begin{array}{c} \text{Corrected EI}=\text{raw EI}+\left(\text{subcutaneous fat thickness }*\;40.5278\right). \end{array}$$

#### 3.1.2. Dynamic Strength Testing

During the first visit, participants performed the dynamic knee extension strength assessment of their dominant leg utilizing a custom-built leg extension device. 1RM testing was performed according to guidelines for strength testing [35] and determined in no more than five maximal attempts.

#### 3.1.3. Resistance Exercise Protocol

The second visit required participants to perform 4 sets of dynamic knee extensions to failure at 50% of the 1RM determined during the first visit. Participants were seated on the knee extension device and performed the repetitions at a controlled pace (50 beats per minute on a metronome) and through the concentric and eccentric phases. To ensure repetitions were completed through the participants full range of motion, a mark was placed parallel to the participants' dominant leg at terminal knee extension. Each successful repetition was required to reach this point, with each set concluding at either the participant's volitional failure or two consecutive attempts of failure to reach terminal knee extension. Two minutes of rest separated each set. The total number of repetitions completed for each set was summed to generate the outcome variable of total repetitions.

#### 3.1.4. Statistical Analysis

SPSS (Version 29; IBM Corp, Armonk, New York) was used for all analyses. Mean and standard deviation were generated to describe maximal strength, total repetitions, and ultrasound-derived measurements of muscle thickness and EI. Bivariate tests, i.e., Pearson correlation, examined the relationships between maximal strength and total repetitions with ultrasound-derived measurements of muscle thickness and EI. Multiple linear regression was run to examine the ultrasound-derived variable from each muscle that demonstrated the greatest association with maximum strength and local muscle endurance. Nine independent variables from three quadriceps muscles were entered stepwise into the model. These included the rectus femoris (1. MT, 2. EI, and 3. cEI), vastus lateralis (4. MT, 5. EI, and 6. cEI), and the vastus intermedius (7. MT, 8. EI, 9. cEI). The modeling was utilized to generate a prediction equation for the 1 repetition maximum and total repetitions. For the 1 repetition maximum strength, independence of residuals was observed by a Durbin–Watson statistic of 1.877. Linearity and homoscedasticity were observed and demonstrated through a visual inspection of a plot of studentized residuals versus unstandardized predicted values. Normality was met following a histogram plot of standardized residuals. There was no multicollinearity as assessed by tolerance (all variables >0.9). No significant outliers were detected by examining the studentized deleted residual and Cook's Distance at a level of ±3 standard deviations. For total repetitions, no variables demonstrated significant relationships and no variables were entered into the model. The strength of the associations is generally interpreted as weak (*r* = 0.10–0.39), moderate (*r* = 0.40–0.69), and strong (*r* = 0.70–0.89) [41]. Alpha was set at 0.05.

## 4. Results

### 4.1. Correlations

**Figure 2:**
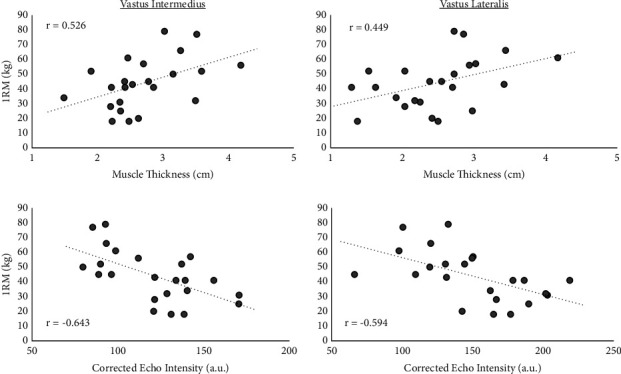
The significant relationships between VI and VL MT and cEI with knee extension 1RM strength.

The associations between each of the knee extensor muscles' ultrasonography outcomes with maximal strength (1RM) and local muscle endurance (total repetitions) are reported in Table 1.  Figure 2 shows the relationships between VI and VL MT and cEI that demonstrate a significant correlation with maximal strength. The muscle fitness and ultrasonography values are reported in Table 2.

### 4.2. Stepwise Multiple Linear Regression

Stepwise multiple linear regression was run to examine how three ultrasound measures scanned in the sagittal plane (MT, EI, and cEI) collected from three knee extensor muscles (VL, VI, RF) predict maximal strength and local muscle endurance during the knee extension exercise. The regression model indicated that VI cEI was the strongest predictor of 1RM strength (*F* (1, 22) = 15.486, *p* < 0.001, *R*^2^ = 0.413, adjusted *R*^2^ = 0.386, and SEE = 14.23 lbs). No variables collected in the current study demonstrated significant predictive ability for the outcome measure of total repetitions.

## 5. Discussion

The current study examines the relationships between skeletal muscle ultrasonography outcomes in the VI, VL, and RF with muscle fitness during knee extension in a habitually endurance-trained population. The main findings demonstrate that ultrasonography outcomes (MT, EI, and cEI) measured in the VI have the strongest relationship with maximal strength, with cEI as the strongest predictor for maximal strength during knee extension. No ultrasonography-derived measures for the VI, VL, or RF demonstrate significant relationships with local muscle endurance. The novel findings of this study demonstrate that ultrasonography measurements in the VI, particularly cEI, are associated with maximal knee extensor strength. These findings may assist ultrasonography imaging considerations when examining muscle fitness in individual quadriceps muscles within sports medicine settings.

Despite the well-established relationship between lower body strength and muscle size of the entire quadriceps [1–6], the examination of muscle size and muscle strength relationships in each of the quadriceps muscles has been limited [38, 42]. In the current study, MT measurements generated from ultrasonography imaging of the VI reported the greatest association with maximal strength (*r* = 0.526; *p*=0.008), followed by the VL (*r* = 0.449, *p*=0.028). These findings are consistent with Strasser et al. [42], who demonstrate that VI MT (*r* = 0.918, *p* < 0.001) has the strongest relationship with knee extensor MVC in young adults. The quadriceps muscles synergistically act on a common tendon during the knee extension action, but have architectural differences that suggest the VI may have greater contributions to maximal knee extension strength [43, 44]. Similarities in the muscle architecture of the superficial quadriceps muscles (RF, VM, and VL) and moderate homogeneity in fascicle length and angle along each muscle's length suggest similarities in their force-generating potential [43]. However, the deep VI is architecturally dissimilar to the superficial quadriceps muscles, with heterogeneity in the fascicle length and angle across the length of the muscle [43]. Novel research using MRI to quantify the mechanical effects of these architectural differences has assessed the *Z*-axis displacement during isometric knee extension [44]. *Z*-axis displacement represents the movement of the muscle along the line of action with the quadriceps muscles and patellar tendon [44]. Interestingly, VI demonstrates a 57% larger mean *Z*-axis displacement than the other quadriceps musculature, leading the authors to suggest it has a greater contribution to mechanical work during knee extension versus the other vasti muscles [44]. Despite the relationship between quadriceps MT and strength, there was no significant association between MT and local muscle endurance. The lack of significant relationships between individual quadriceps muscle size and performance may be explained in part by the synergistic contributions of each muscle during knee extension [43, 44]. It may also be that the relationships between muscle size and local muscle endurance seen for isometric tasks are explained by size-dependent intramuscular pressures and metabolite accumulation [45–48]. Overall, the findings suggest that VI MT has a substantial influence on maximal knee extension strength in endurance-trained individuals.

While VI MT demonstrates a significant, positive relationship with maximal strength (*r* = 0.526, *p*=0.008), VI cEI (*r* = −0.643, *p* < 0.001) demonstrates the greatest predictive ability for maximal strength in the current study. Echo intensity demonstrates a negative relationship with maximal strength during knee extension in older adults, measured in the VM [29, 30], the RF [25–27], and the VI [28]. However, examinations in younger populations are less common, with Mota and Stock [31] reporting a nonsignificant, moderate relationship between quadriceps EI and maximal strength (*r* = −0.56, *p*=0.06) and Yoshiko et al. [32] reporting a significant negative relationship between quadriceps EI and maximal strength (*r* = −0.61, *p* < 0.001). Consistent with the current study, Yoshiko et al. [32] reported stepwise multiple linear regression identified quadriceps EI (averaged from the RF and VL) as the strongest predictor of maximal knee extension strength in young, untrained individuals rather than MT. Thus, the unexplained variance between muscle size and strength may be explained in part by the concentrations of contractile and noncontractile tissue in a muscle quantified by EI. Echo intensity appears to be related to the intramuscular fat content measured via MRI [20]. Increases in fat accumulation associates with lower force development [23, 24], impairments in Ca^2+^ release from the sarcoplasmic reticulum [49], and lower central activation [50]. In addition to intramuscular fat concentrations, increases in hyperechoic intramuscular fibrous tissue may occur as aging progresses [25, 31]. These changes may produce a greater EI value and suggest decreased muscle quality [18] in older or young adults. However, the negative relationship between EI and maximal strength is maintained in the young, habitually endurance-trained population of the current study and in habitually resistance-trained individuals [7]. The lack of consensus and limited evidence for the effect that training has on EI [17, 31, 51] provides an opportunity to explore the use of EI as a muscle fitness predictor in trained populations.

Trained individuals are hypothesized to present with lower EI due to an increase in hypoechoic muscle fiber size relative to the volume of hyperechoic perimysium tissue [52–55]. In addition, the increase in muscle fiber size produces a greater volume of tissue that an ultrasonographic signal is required to penetrate. This may decrease the EI of deep muscles and artificially decrease EI when averaging EI across multiple muscle groups. This may be similar to the hypoechoic effect adipose tissue presents on underlying tissue [7, 18, 20]. However, endurance exercise may increase the reported EI value. The preferentially utilized type I muscle fibers demonstrate greater oxidative capacity in addition to greater intramuscular fat concentrations surrounding their muscle fiber bundles and lipid content, possibly increasing the EI value [33, 34]. Alternatively, increased hypoechoic capillarization within the muscle may decrease EI [52–55] and suggest a dynamic interplay on the EI value in endurance-trained individuals. In the current study, EI demonstrates no significant relationships with total knee extension repetitions performed at 50% 1RM. Similarly, Mota and Stock [31] observed no associations between quadriceps EI and local muscle endurance in young and older men performing 50% 1RM knee extensions. In older adults, quadriceps EI is not associated with cycle ergometer maximal oxygen uptake (VO_2_ max) during cycle ergometry [25]. However, EI is positively associated with total bicep curl repetitions performed at 50% 1RM in resistance-trained individuals [7]. As multiple muscles contribute to knee extension, EI obtained from individual muscles of the quadriceps may not have significant relationships or predictive ability with local muscle endurance as observed in the biceps curl wherein the biceps is the prime mover [7]. In addition, the muscle-specific EI value may not represent whole-body cardiorespiratory adaptations to habitual endurance training contributing to local muscle endurance performance. Further research is encouraged to examine the relationship between EI and local muscle endurance in the prime movers of a task. The opportunity exists to further EI research by examining muscle endurance adaptations to training in conjunction with EI. This may further expand the current knowledge of musculoskeletal composition and its influence on the EI measure.

### 5.1. Limitations

The current study has limitations for consideration. The individuals in the current study reported habitual endurance exercise, but heterogeneity in training may have altered individual muscle fitness relationships with knee extension performance. If participants performed resistance training, which was not quantified in the current study, knee extension performance may have been increased. However, all participants met the criteria of ∼300 minutes per week of moderate to vigorous aerobic activity over the last 12 months and may have similar physiological training adaptations. In addition, the use of a traditional, dynamic constant external resistance device provides a constant training intensity rather than an accommodating resistance and omits information related to the force-time curve during the work bout, information that may be obtained with isokinetic dynamometry. However, we contend the use of a pragmatic piece of resistance exercise equipment enhances the translation of the current findings to sports medicine settings and practitioners. Of the four main quadriceps muscles, only the VI, VL, and RF were examined. While each muscle contributes to knee extensor performance [43, 44, 56], the VM is the second smallest quadriceps muscle [43] and is suggested to have the smallest contribution to knee extensor performance of the four quadriceps muscles [43, 56]. Thus, its exclusion may not have influenced the findings in the current study. In addition, image acquisition was performed solely in the sagittal plane. Image acquisition may be performed utilizing an extended field of view, or panoramic, to obtain muscle cross-sectional area. However, prior work from our laboratory examining the effect of the sagittal, transverse, and extended field of view ultrasonography scanning planes and their relationships with muscle fitness demonstrates the sagittal plane has the strongest relationship with measures of muscle fitness in the upper limb [7]. Imaging of the quadriceps muscles was performed at a singular point that corresponded with the location of greatest cross-sectional area for the VI, VL, and RF [38]. This methodological decision limits the investigation of volumetric measures of muscle size along with regional differences in the quadriceps muscles. However, as muscle thickness was utilized to examine muscle size, the respective area with the greatest cross-sectional area provides confidence to our investigations of maximal muscle thickness with maximal strength and local muscle endurance. It is important to note the lack of exact foundational premise of the EI measure. Increases in EI are associated with increased intramuscular adiposity [18–20], but changes in EI following training are uncertain [57]. In addition, methodological approaches during acquisition such as probe tilt, ultrasound settings, or acquisition and analysis experience [18, 37] may alter EI values. Similarly, heterogeneity in participant characteristics such as skin color [58–60] or adipose tissue thickness [7, 18, 20] may confound the reported EI. Therefore, training was performed [37] and the population in the current study did not vary considerably in skin pigmentation, minimizing these influences.

## 6. Conclusion

This current study provides important considerations for the use of ultrasonographic measures to examine muscle fitness in habitually endurance-trained populations. The findings demonstrate a moderate to strong relationship between VI and VL MT, EI, and cEI with maximal strength during the knee extension task. Of the individual quadriceps muscles investigated, the current study demonstrates that cEI of the VI is the strongest predictor of maximal knee extensor strength. It is interesting that cEI, rather than MT, was a predictor of maximal strength. Muscle architectural differences within the quadriceps may confound the relationship between muscle size and strength, increasing the importance of muscle quality on maximal strength production. Therefore, examinations of EI in healthy, trained populations may provide additional information about muscle fitness in the knee extensors than muscle thickness alone. The findings suggest that monitoring VI muscle size and quality may benefit performance and rehabilitation when knee extension is a required skill. The findings in the current study present the use of ultrasonography to examine individual quadriceps muscle fitness and strengthen the implementation of EI measurements in trained populations.

## Figures and Tables

**Table 1:** Correlation of quadriceps muscles ultrasonography measures with maximal strength and local muscle endurance.

Measure	1 repetition maximum		Total repetitions	
	Pearson correlation	*p* value	Pearson correlation	*p* value
Rectus femoris				
Muscle thickness	0.299	0.155	0.120	0.576
Echo intensity	0.049	0.820	−0.130	0.545
Corrected EI	−0.400	0.053	−0.130	0.546
Vastus intermedius				
Muscle thickness	0.526	0.008^*∗*^	−0.105	0.624
Echo intensity	−0.446	0.029^*∗*^	0.156	0.466
Corrected EI	−0.643	<0.001^*∗*^	0.049	0.822
Vastus lateralis				
Muscle thickness	0.449	0.028^*∗*^	0.076	0.723
Echo intensity	−0.368	0.077	0.120	0.577
Corrected EI	−0.594	0.002^*∗*^	0.040	0.851

^*∗*^Denotes significant correlation at the *p* < 0.05 level.

**Table 2:** Muscle fitness and ultrasonography outcomes.

Measure	Mean ± SD
1 repetition maximum	44 ± 17.3 kg
Total repetitions	71 ± 22
Muscle thickness	
Rectus femoris	2.60 ± 0.50 cm
Vastus intermedius	2.71 ± 0.62 cm
Vastus lateralis	2.48 ± 0.70 cm
Echo intensity	
Rectus femoris	120.78 ± 22.33 a.u.
Vastus intermedius	69.80 ± 12.76 a.u.
Vastus lateralis	112.21 ± 21.46 a.u.
Corrected echo intensity	
Rectus femoris	123.16 ± 26.03 a.u.
Vastus intermedius	121.34 ± 26.92 a.u.
Vastus lateralis	149.79 ± 38.17 a.u.

## Data Availability

The data used to support the findings of the study are available upon reasonable request from the corresponding author.
